# Design of Compressed Sensing Algorithm for Coal Mine IoT Moving Measurement Data Based on a Multi-Hop Network and Total Variation

**DOI:** 10.3390/s18061732

**Published:** 2018-05-28

**Authors:** Gang Wang, Zhikai Zhao, Yongjie Ning

**Affiliations:** 1 The National Joint Engineering Laboratory of Internet Applied Technology of Mines, Xuzhou 221000, China; wanggang@cumt.edu.cn; 2 IOT Perception Mine Research Center, China University of Mining and Technology, Xuzhou 221000, China; TS16060245P3@cumt.edu

**Keywords:** compressed sensing, moving measurement data, multi-hop network, total variation, coal mine

## Abstract

As the application of a coal mine Internet of Things (IoT), mobile measurement devices, such as intelligent mine lamps, cause moving measurement data to be increased. How to transmit these large amounts of mobile measurement data effectively has become an urgent problem. This paper presents a compressed sensing algorithm for the large amount of coal mine IoT moving measurement data based on a multi-hop network and total variation. By taking gas data in mobile measurement data as an example, two network models for the transmission of gas data flow, namely single-hop and multi-hop transmission modes, are investigated in depth, and a gas data compressed sensing collection model is built based on a multi-hop network. To utilize the sparse characteristics of gas data, the concept of total variation is introduced and a high-efficiency gas data compression and reconstruction method based on Total Variation Sparsity based on Multi-Hop (TVS-MH) is proposed. According to the simulation results, by using the proposed method, the moving measurement data flow from an underground distributed mobile network can be acquired and transmitted efficiently.

## 1. Introduction

During the application of both a coal mine Internet of Things (IoT) mine and many mobile measurement devices (e.g., intelligent miner’s lamp), a large amount of moving measurement data is generated, and transferring these amounts of monitoring data efficiently is a challenge. The moving measurement data in mine IoT include mobile gas measurement data, mine equipment multi-channel measurement data, distributed mine monitoring data and mobile target location data. Different types of mobile data transmission and processing technologies have similarities. This paper presents a compressed sensing algorithm for coal mine moving measurement data based on a multi-hop network and total variation by taking gas data in the mobile measurement data as an example.

Underground gas is a kind of gas resource stored in coal and rocks; it gushes out during the mining of coal and causes a gas outburst under certain conditions. Gas explosion poses grave threats to coal’s safe production. The coal mine safety monitoring system needs to monitor the environmental parameters such as gas concentrations, wind speed, dust concentrations and so on. The traditional gas sensors are basically fixed-point detection sensors, so the amount of monitoring data is not great. With the development of mine IoT, a great number of intelligent miner’s lamps are put into use. For example, in a large mine, there are approximately a thousand people who work underground every day. The miner’s lamp and cap are necessary for workers to work underground and gas sensors are in the cap and move with the miners. It can be regarded as a mobile measurement system for underground gas. Compared with the fixed gas sensors, the application of the mobile gas sensors not only expands the monitoring range, but also can obtain the variation curve of gas with time in different positions along the coal mine roadway. In a smart cities environment, a fog-supported smart city network (FOCAN) architecture for management of everything is proposed [1], which can provide services with low energy usage. Habib et al. studied the barrier coverage in wireless sensor networks for intrusion detection and border surveillance [2]. The network in an underground coal mine is an industrial Ethernet, which is a relatively closed network, and the underground access users are authorized users. Miners work underground for no more than 10 h, and every miner’s lamp will be fully recharged before he goes down the mine, so energy conservation is not the main problem. At the same time, the coal mine roadway is a long linear structure, which makes the network structure different from that in ground application.

As intelligent miner’s lamps became widespread in mines, the amount of moving measurement data increased dramatically. How to achieve high-efficiency acquisition and transmission of gas data flow appears to be an urgent problem that needs to be solved for underground distributed mobile gas detection systems.

For scalable distributed in-network computation, data compression proves to be an effective solution for achieving high-efficiency transmission and storage of gas monitoring data. The SensorScope project undertaken by the Lausanne Federal Institute, Switzerland, used a wireless sensor network for monitoring environmental information [3]. Yin et al. developed a gas data compression method for gas time series based on prediction and run-length coding [4]. To overcome the inefficiency of intelligent algorithms induced by the high-dimensional characteristics of coal gas data, Zhao et al. proposed a graph-based parameter-free dimensionality reduction algorithm and a locality-preserving semi-supervised dimensionality reduction algorithm [5]. A sub-band energy adaptive data compression (SEADC) method in wireless network was proposed by Huang et al. [6]. According to the data-streaming characteristics in the sensor network, Xie et al. proposed a mixing entropy data compression based on interval wavelet transformation to achieve the compression on streaming data in the sensor network [7]. Ziv et al. presented a universal algorithm for sequential data compression through which performance is investigated with respect to a non-probabilistic model of constrained sources [8]. How to make use of the correlation between mobile gas data and realize the simultaneous recovery of multiple measurement nodes with a small number of measurement data still needs to be studied.

Compressed sensing takes full advantage of the signal’s sparsity and can perform non-adaptive measurement encoding on the signal at a far lower sampling rate than the Nyquist sampling rate. Due to the novelty in its theoretical framework, compressed sensing now has provided some new ideas for many practical signal processing problems [9]. Sparse recovery algorithms can be classified into three main categories: Convex and Relaxation, Greedy, and Bayesian [10]. In the application of data monitoring based on Wireless Sensor Network, Xue put forward multiple access and data reconstruction in wireless sensor networks based on compressed sensing [11]. Uthayakumar et al. presented a data compression algorithm to maximize network lifetime in wireless sensor networks [12]. Lossy transmission is a common problem for monitoring systems based on wireless sensors; Zou et al. presented a data loss recovery technique based on compressed sensing to enhance communication reliability [13].

In view of the long linear structure of coal mine roadways and the time sparsity of gas sensors, a compression and reconstruction algorithm for a coal mine network transmission structure is studied in this paper. The algorithm takes advantage of the time correlation of the mobile gas monitoring data in multi-hop networks and realizes the sparsity of monitoring data by introducing total variation. By studying the relationship between data transmission and compressed sensing in multi-hop networks, a Total Variation Sparsity based on Multi-Hop (TVS-MH) algorithm is proposed, and the effectiveness of the algorithm is verified. The rest of this paper is organized as follows. Section 2 introduces the model of the mine IoT monitoring system, which is a mixed network of wired and wireless networks. Section 3 introduces the basic theory of compressed sensing, and a compressive acquisition model based on compressed sensing for gas data flow is presented in Section 4. Then, in Section 5, a high-efficiency gas data compression and reconstruction method TVS-MH is proposed. Section 6 presents gas data reconstructed based on TVS-MH. A series of tests show that the moving measurement data flow from an underground distributed mobile network can be acquired and transmitted efficiently by using the proposed method. Finally, conclusions are drawn in Section 7.

## 2. Model of the Mine IoT Monitoring System

**Figure 1:**
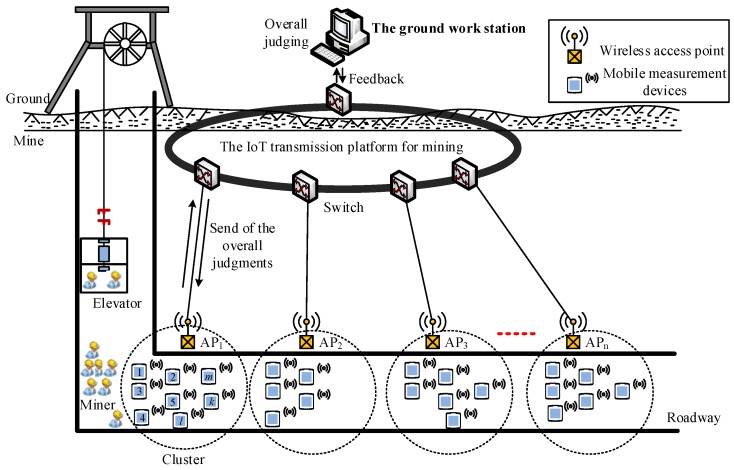
Model of the mine IoT monitoring system.

Fog-supported smart city architecture can support several heterogeneous devices to connect to each other [1], but it is slightly complicated for the monitoring environment of mine gas. The moving measurement data monitoring network based on IoT architecture is illustrated in Figure 1. A wireless access point with basic pre-analysis and judging programs for mobile measurement data was attached to a switch through a wired connection. When the miners are transported to the underground by the elevator, there may be several miners passing through the same access point at the same time. As the miners with smart safety lamps that are equipped with a portable sensor passed by a wireless access point, the data is pre-analyzed and successively transmitted to the ground work station. Because the mobile measurement points measure the gas data at the same time and in the same space, the data have great correlation. As we know, compressed sensing takes full advantage of the signal’s sparsity, and can be constructed to reduce the amount of transmission data. 

## 3. Theoretical Framework of Compressed Sensing

### 3.1. Theoretical Framework

**Figure 2:**
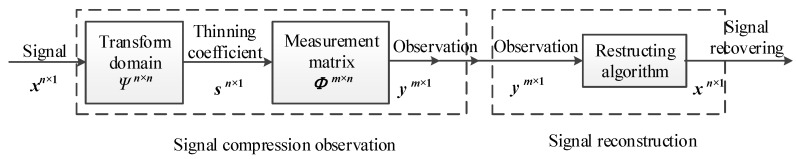
Compression sensing code block diagram.

Traditional signal acquisition and processing includes four steps: sampling, compression, transmission, and decompression. The sampling process should satisfy Shannon’s sampling theorem. Compressed sensing performs signal sampling and compressive encoding in a step. As illustrated in Figure 2, compressed sensing takes full advantage of the signal’s sparsity and conducts non-adaptive measurement and encoding on the signal at a sampling rate far lower than the Nyquist sampling rate. 

Compressed sensing includes the following three steps [9]:

(1) The original signal *x*, with a length of *N*, is sparse or sparse under the base of Ψ(N×N), and the sparse signal is denoted as *S*;
(2) The observed value *y* was acquired based on the observation matrix Φ(M×N),M<<N;
(3) Based on the given Φ, Ψ and *y*, an appropriate algorithm was selected for the recovery of *x*.

Accordingly, compressed sensing includes the sparse representation of a signal, the design of the measurement matrix, and the reconstruction algorithm. The sparse representation of a signal is the prerequisite; the measurement matrix is the means of the structured representation of the signal; and the reconstruction algorithm serves as the guarantee of signal reconstruction.

### 3.2. Sparse Representation of the Signal

For a signal, if only a few of the elements are nonzero, it can be considered sparse. Generally, natural signals in the time domain are non-sparse but can be sparse in some transform domains. These signals require sparse representation. Signal’s sparse representation is a concise representation; specifically, when the signal is projected into the orthogonal transform basis, most of the transformation coefficients indicate small absolute values and the acquired transformation vectors are sparse or approximately sparse. This is also the priori condition of compressed sensing, i.e., the signal must be represented in a sparse manner under certain transformations.

A one-dimensional discrete time signal with a length of *N* can be expressed as the following linear combination of a set of orthogonal bases:

(1) $$x=\sum_{i=1}^{N}s_{i}\psi_{i}\;\mathrm{or}\;x=\Psi s$$

where $\Psi =[\psi_{1},\psi_{2},\cdot \cdot \cdot ,\psi_{N}]$ ($\psi_{i}$ denotes the *i*-th column vector). Apparently, ***s*** is an equivalent representation of *x*. If s only includes few great coefficients, then x is compressible; if *s* only has *K* nonzero elements, then s can be regarded as a *K*-sparse representation of the signal *x* [14].

In general applications, the transformation basis can be set as flexible according to signal characteristics. Some commonly used transformation bases include the discrete cosine transform (DCT) basis, the fast Fourier transform (FFT) basis, the discrete wavelet transform basis [15], the Curvelets basis [16], and the Gabor basis [17]. For signals that cannot be represented using orthogonal bases, sparse representation can be conducted by means of a redundant dictionary [18,19].

### 3.3. Measurement Matrix

Given a *K*-sparse signal *x* with a length of *N*, and a measurement matrix $\Phi \in {\mathbb{R}}^{M\times N}(M<<N)$, we then calculated the measured value. If *x* is sparse, then the measured value could be calculated by y=Φx, $y_{j}=\langle x,\phi_{j}\rangle$; if *x* is non-sparse, then we first performed sparse representation x=Ψs and then calculated the measured value, y=Φx=ΦΨs=Θs. Each row of Φ can be regarded as a sensor. Part of the information can be picked up by multiplying the signal by Φ. 

To achieve the reconstruction of signal, Candès and Tao proposed and proved that the sensing matrix Θ should satisfy the restricted isometry property [20]. For any *K*-sparse signal *v* and a constant $\delta_{K}\in (0,1)$, if

(2) $$(1-\delta_{K}){\left\|v\right\|}_{2}^{2}\leq {\left\|\Theta v\right\|}_{2}^{2}\leq (1+\delta_{K}){\left\|v\right\|}_{2}^{2}$$

is satisfied, then the matrix satisfies the restricted isometry property. Candès et al. proved that if Φ is a Gaussian random matrix, then the sensing matrix Θ can most likely satisfy the restricted isometry property [21]. Therefore, we can select a Gaussian measurement matrix, with a size of M×N, to construct Φ, in which each value satisfies the independent normal distribution of N(0,1/N). Some other measurement matrices that can make the sensing matrix satisfy the restricted isometry property include the consistent spherical/ball matrix, the two-value/binary random matrix, the local Fourier matrix, the local Hadamard matrix and the Toeplitz matrix [22].

### 3.4. Reconstruction Algorithm

A signal reconstruction algorithm is the core of compressed sensing theory and refers to the reconstruction of the sparse signal *x* with a length of *N* from the measurement vector *y* with a length of M. Since y=Φx and the dimension of *y* is far smaller than that of *x*, the equation has infinitely many solutions and the signal cannot be reconstructed. However, if the original signal is *K*-sparse and the measurement matrix satisfies certain conditions, the signal *x* can be accurately reconstructed from the measured value *y* through the solution of the $l_{0}$ norm. The reconstruction was proved theoretically and can be written as follows:

(3) $$\hat{x}=\arg\;\min\;{\left\|x\right\|}_{0}\;s.t.\;\Phi x=y$$

where ${\left\|\cdot \right\|}_{0}$ denotes the vector’s $l_{0}$ norm, which is the number nonzero elements in the vector *x*. 

## 4. The Compressive Acquisition Model for Gas Data Flow

Compressive-sensing-based signal compression, acquisition and recovery has the following characteristics.

(1) Encoding end is simple: only a measurement matrix that follows random distribution and satisfies incoherence is required and codes are non-adaptive.
(2) Encoding and decoding are unequal to each other: at the encoding end, the computation is simple, indicating less energy consumption and uniqueness; at the decoding end, the computation is quite complex, and many reconstruction algorithms can be selected.
(3) Compressed sensing is characterized by robustness and noise immunity. Since the measurement matrix has allotted high-dimensional signal information to each measured value, each measurement coefficient indicates the same importance or unimportance and the errors caused by the loss of several measurement coefficients are the same.

Therefore, compressed sensing is particularly applicable to signal processing systems that require low-energy-consumption sensor encoding but have high computing capability at the decoding end. The gas data detected by a mobile gas detection instrument were transmitted to wireless access points via a wireless network; when multiple mobile gas detection instruments moved underground, the mobile gas detection instruments near a wireless access point constituted a wireless sensing network. The mobile gas detection instruments were poor in data processing, but the principal computer exhibited extremely strong signal processing capability; therefore, the mobile gas detection instruments constituted a system that is exactly suitable for the compressed sensing theory.

### 4.1. Two Different Networks for the Transmission of Gas Data Flow

Data between two wireless access points can be transmitted through two different types of networks, namely the single-hop network and the multi-hop network. These two networks have different data transmission modes. This paper assumes that different routing protocols can be completed through selected routing protocols.

#### 4.1.1. The Single-Hop Mode

**Figure 3:**
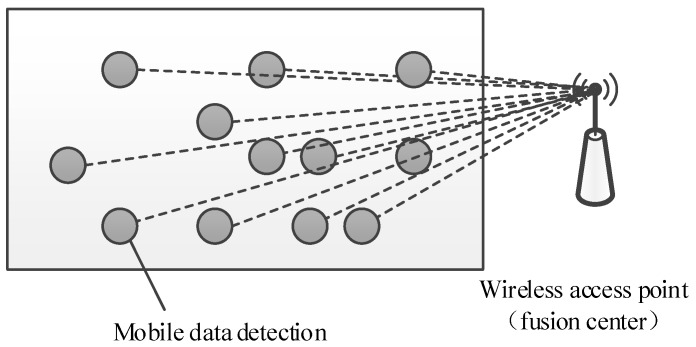
Single-hop wireless networks.

As illustrated in Figure 3, the current study assumed that all mobile gas detection instruments were within the communication range of wireless access points, periodically measured the environmental gas data and directly sent the data to wireless access points (fusion centers).

Assume that *N* denotes the number of mobile gas detection instruments and $x_{j}$ denotes the gas data measured by the *j*-th sensor. According to the requirements of compressed sensing theory, the measured gas data by each mobile gas detection instrument at *M* time intervals were multiplied by *M* random coefficients ${\left\{\Phi_{i,j}\right\}}_{i=1}^{M}$, and the products were then transmitted to wireless access points. The random coefficients were generated by setting the ID of the mobile gas detection instrument as the seed of the pseudo-random sequence generator. At the *i*-th time interval, *N* mobile gas detection instruments simultaneously transmitted $\Phi_{i,j}x_{j}$ to wireless access points; therefore, the signal received by the wireless access point at the i-th time interval can be written as [23,24]:

(4) $$y_{i}=\sum_{j=1}^{N}\Phi_{i,j}x_{j}$$

At each time interval, the signal received by the wireless access point is the sum of all the *N* measurements of mobile gas instruments multiply the *N* random coefficients ${\left\{\Phi_{i,j}\right\}}_{j=1}^{N}$, so the wireless access point is also called fusion center. 

At the end of *M* time intervals, the signal vector received by wireless access points can be written as: $y={\left[y_{1},\cdots ,y_{M}\right]}^{T}$. In a matrix form, y can also be rewritten as follows:

(5) y=Φx

where Φ denotes an *M* × *N* matrix. 

Although the transmitted gas vector x is not sparse, gas vectors can be regarded to be sparse in some transform domains due to the spatial correlation of physical phenomena. By assuming the gas vector x has a decomposition coefficient *s* under certain orthogonal bases, and *s* is sparse, the following expression can be acquired:

(6) y=Φx=ΦΨs

It can thus be transformed into a standard compressed sensing problem.

#### 4.1.2. The Multi-Hop Mode

**Figure 4:**
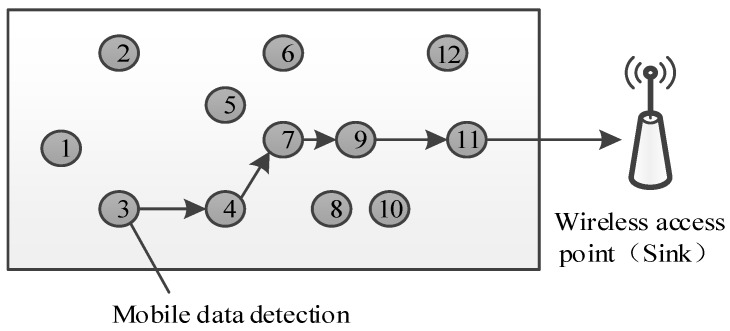
Multi-hop wireless networks.

As illustrated in Figure 4, in addition to the single-hop mode, data can be transmitted through a multi-hop mode when multiple mobile gas detection instruments move between two wireless access points. Data were aggregated as follows. 

Data were acquired though *M* time-independent multi-hop communication links with different initial nodes. Each multi-hop network randomly selected an initial node. At each node, the measured gas value was multiplied by the generated random coefficient; then, the product was transmitted to the next node according to the preset routing algorithm. At the next node, the received signal was added to the local product, and the sum was transmitted to the next node. By analogy, the *i*-th multi-hop signal received by the wireless access point can be written as [25]:

(7) $$y_{i}=\sum_{j\in P_{i}}\Phi_{i,j}x_{j}$$

where $P_{i}$ denotes the node index set of the *i*-th multi-hop communication link, $\Phi_{i,j}$ denotes the random coefficient at the *j*-th node, and $x_{j}$ denotes the gas data measured at the *j*-th node. Similarly, based on the spatial correlation of the measured gas data, the signal vector received by the wireless access point $y=[y_{1},\cdots ,y_{M}]$ can be written as follows:

(8) y=ΦΨs

where Φ denotes an *M* × *N* matrix (when $j\in P_{i}$, the element at the *i*-th row and the *j*-th column equaled $\Phi_{i,j}$; otherwise, equaled 0). Accordingly, it can be regarded as a standard compressed sensing problem.

### 4.2. Compressive Acquisition Model of Gas Data Flow

In the current study, a mobile gas detection instrument was mounted on a miner’s lamp, in which the GS1011 low-power consumption Wi-Fi chip was the hardware core. Since the miner’s lamp took multiple functions (sensing, lighting, and energy communication), low energy consumption of the mobile gas detection instrument should be an important constraint. Considering the lamp’s limited energy, the multi-hop communication mode was preferable when several mobile gas detection instruments moved between two wireless access points. 

**Figure 5:**
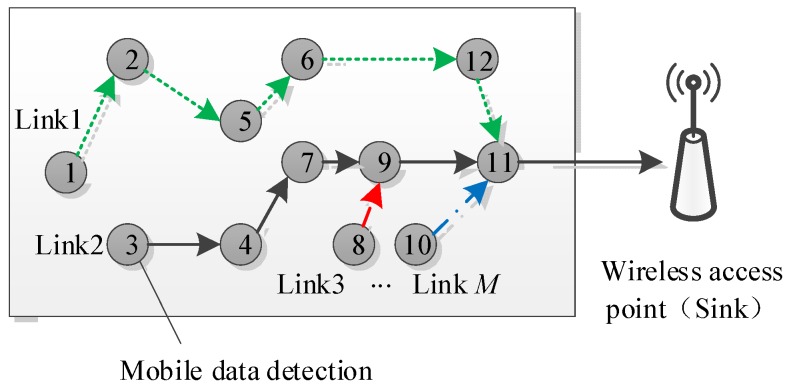
Compression collection model of gas flow data.

Accordingly, the signals on *M* time-independent multi-hop communication links with different initial nodes should be collected. As illustrated in Figure 5, by assuming that $y_{i}=\sum \Phi_{i,j}x_{j}$ denotes the *i*-th multi-hop signal received by a wireless access point (i=1,2⋯M), in which $\Phi_{i,j}$ denotes the random coefficient generated at the *j*-th node in the *i*-th multi-hop communication link and $x_{j}$ denotes the gas data measured at the *j*-th node, the receiving vector of *M* multi-hop communication links was denoted as $y=[y_{1},\cdots ,y_{M}]$, i.e., y=ΦΨs.

Selection of measurement matrix is a critical step in the practical application of compressive sensing theory. The related theory has proved that, when M≥CKlog(N/M), all *K*-sparse vectors can be stably recovered from some random measurement matrices such as Gaussian matrix, Bernoulli matrix or Fourier random matrix. However, if a random matrix was generated in each measurement and recorded for finally reconstruction, the system cost would be increased in practical applications, which is not beneficial for physical implementation. Therefore, some mathematicians proposed to use deterministic measurement matrix and structured measurement matrix to replace the random matrix. Some theoretical and experimental results also proved that structured measurement matrix is an effective choice. In the proposed compressive acquisition model, the communication links are generated according to the adopted multi-hop route which is determined, and the coefficient generated at the *j*-th node in the *i*-th multi-hop communication link is random, so the Φ is pseudorandom. It satisfies the RIP properties [26].

## 5. Gas Data Reconstructed Based on TVS-MH

Reconstruction of gas data is the core of compressed sensing of gas data flow. It can be theoretically proved that, if the gas data are *K*-sparse and the measurement matrix satisfies certain conditions, the signal *x* can be accurately reconstructed from the measured value of *y* by solving the $l_{0}$-norm problem:

(9) $$\hat{x}=\arg\;\min\;{\left\|x\right\|}_{0}\;s.t.\;\Phi x=y$$

However, Donoho pointed out that the minimum $l_{0}$-norm problem is also an NP-hard problem and all possible arrangements of non-zero values in ($C_{N}^{K}$) should be exhausted. Therefore, scholars proposed a series of algorithms for near-optimal solutions. Gas signals are generally slowly varying and only exhibit occasional rapid variations at mutation points, and the mobile measurement points measure the gas data at the same time and in the same space; this means that $x_{i}\approx x_{i+1}$ in most cases. Therefore, the total variation of $x\in R^{N}$ can be defined as follows:

(10) $$||Dx|{|}_{1}=\sum_{i=1}^{N-1}\left|D_{i}x\right|=\sum_{i=1}^{N-1}\left|x_{i+1}-x_{i}\right|$$

where $D\in R^{(N-1)\times N}$ is a dual-diagonal matrix, and $D_{i}$ denotes the element in the *i*-th row of *D*. *D* can be written as follows:

(11) $$D=\left[\begin{array}{ccccccc} -1 & 1 & 0 & \cdots & 0 & 0 & 0\\ 0 & -1 & 1 & \cdots & 0 & 0 & 0\\ \vdots & \vdots & \vdots & \ddots & \vdots & \vdots & \vdots \\ 0 & 0 & 0 & \cdots & -1 & 1 & 0\\ 0 & 0 & 0 & \cdots & 0 & -1 & 1 \end{array}\right]$$

The total variation function assigned larger values to rapidly-varying *x*. Accordingly, the reconstruction problem can be rewritten as follows:

(12) $$\underset{x}{\arg\min}||Dx|{|}_{1},\;s.t.\;\Phi x=y$$

To acquire the minimum, the following augmented Lagrangian function was introduced:

(13) $$L(x,\lambda ,\mu )=||Dx|{|}_{1}-\lambda^{T}(\Phi x-y)+\frac{\mu }{2}{\left(\Phi x-y\right)}^{T}(\Phi x-y)$$

Equation (13) can be solved using an iterative algorithm, and the unknown parameters λ and μ can be selected according to the following Equations (14) and (15):

(14) $$\lambda^{k+1}=\lambda^{k}-\mu^{k}(\Phi x^{k+1}-y)$$

(15) $$\mu^{k+1}\geq \mu^{k}$$

where the superscript *k* denotes the *k*-th iterated value.

In contrast to the standard Lagrangian function, the augmented Lagrangian function included a quadratic penalty function.

Algorithm 1 presents the detailed procedures of data reconstruction using the TVS-MH algorithm. 


**Algorithm 1. The TVS-MH Algorithm.**
**1. Input:**$x_{j}$, $\Phi_{i,j}$, i=1,2⋯M, j=1,2⋯N.**2. Step1:** Select *M* time-independent multi-hop communication links with different initial nodes in a cluster**3. Step2:** Assign the *i*-th multi-hop signal received by the wireless access point as $y_{i}=\sum \Phi_{i,j}x_{j}$ (i=1,2⋯M).**4. Step3:** Calculate all of the *M* multi-hop communication links to form a gas data compressed sensing collection model y based on the multi-hop network**5. Step4:** Calculate the total variation of x:6. $||Dx|{|}_{1}=\sum_{i=1}^{N-1}\left|D_{i}x\right|=\sum_{i=1}^{N-1}\left|x_{i+1}-x_{i}\right|$.**7. Step5:** Solve the reconstruction problem that can be rewritten as follows:8. $\underset{x}{\arg\min}||Dx|{|}_{1},\;s.t.\;\Phi x=y$.

## 6. Discussion

In this section, the relationships between data reconstruction accuracy using TVS-MH as well as the signal compression ratio and the reconstruction performances of the TVS-MH algorithm for different types of gas signals were tested; additionally, the reconstruction performances and computation complexities using different signal reconstruction algorithms were compared. 

### 6.1. Comparison of Reconstruction Performances and Computation Complexities of the TVS-MH Algorithm for Different Gas Signals

#### 6.1.1. Gas Mutation States

**Figure 6:**
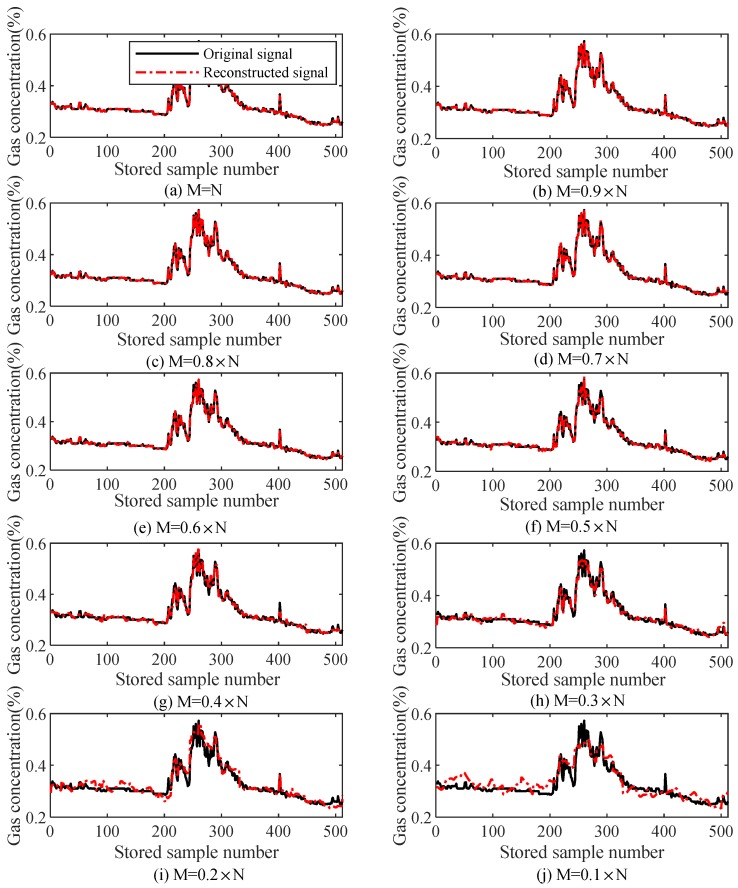
Compression perception and the reconstruction error of gas mutation data (TVS-MH).

Figure 6 illustrates the reconstruction performances under gas mutation states when different numbers of measuring points were used (*M* = 0.1 N~N), during which the signal was reconstructed using the TVS-MH algorithm. The initial parameters were set below.

The first penalty factor was set as: $\mu^{0}=2^{8}$.

The error for stopping the algorithm was set as: $tol=10^{-6}$.

The maximum iteration number was set as 300.

It can be observed that when M≥0.3 N, the gas signal can be accurately reconstructed using the TVS-MH algorithm. In a measurement curve, the reconstructed signal can well reflect the variation of the original signal in both gas mutation states and steady states. In particular, the reconstructed signal can track the mutation segments in original signals well, suggesting TVS-MH algorithm’s favorable reconstruction performances for different types of signals.

Table 1 presents the relations of *M* with the reconstruction error and the interaction number (i.e., the computation complexity) during the reconstruction of gas mutation data using TVS-MH algorithm. It can be observed that, when the compression ratio is greater than or equal to 0.3, the reconstruction error is smaller than 4%, and the iteration number is smaller than 100, suggesting that the TVS-MH algorithm was relatively simple in computation and is thus convenient for applications in practical engineering.

#### 6.1.2. Gas Steady States 

**Figure 7:**
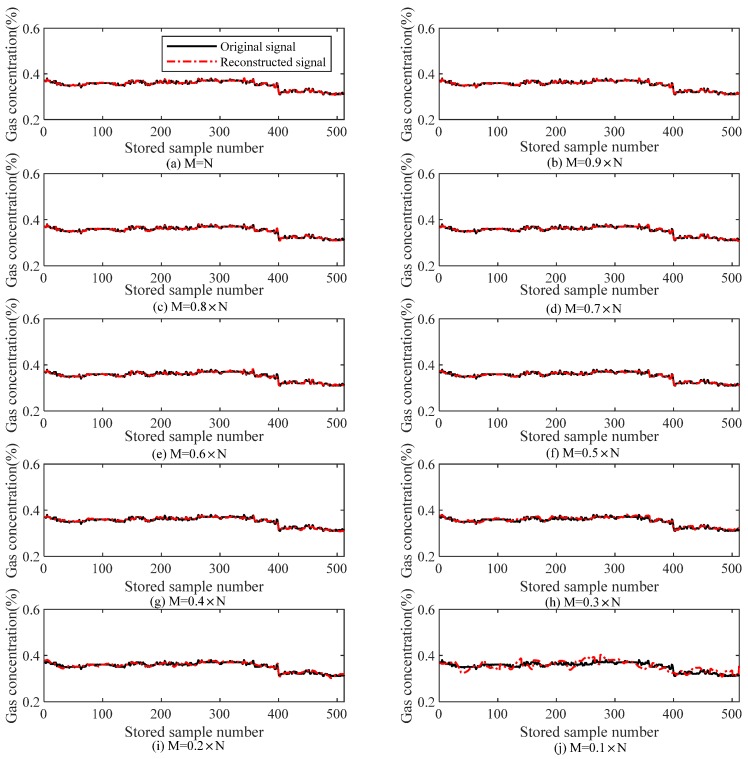
Compression perception and the reconstruction error of the gas steady signal (TVS-MH).

Table 2 lists the relations of *M* with the reconstruction error and the iteration number (i.e., the computation complexity) during the reconstruction of gas data under steady states, and Figure 7 illustrates the effect of the number of measuring points (*M*) on reconstruction performance under gas steady states. It can be observed that since the gas signal varies steadily, the reconstruction accuracy exceeds the value under gas mutation states at the same compression ratio. When the compression ratio is equal to or greater than 0.3, the reconstruction error is smaller than 1.3% and the iteration number is smaller than 100. 

#### 6.1.3. Complex Gas Signal

**Figure 8:**
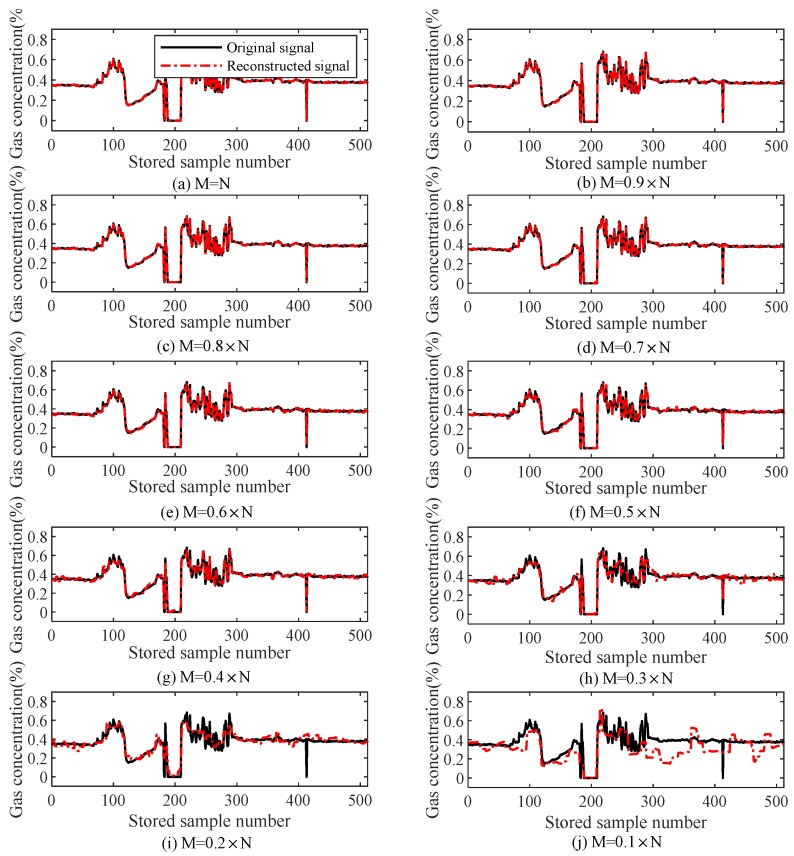
Compression perception and the reconstruction error of the complex gas signal (TVS-MH).

Table 3 lists the relationships of *M* with the reconstruction error and iteration number (i.e., the computation complexity) during the reconstruction of gas data using the TVS-MH algorithm, and Figure 8 illustrates the effect of the number of measuring points (*M*) on the reconstruction performance of a complex gas signal. It can be observed that since the gas signal varies intensively, the reconstruction accuracy is lower than that under the gas mutation state at the same compressive rate. When the compression ratio is equal to or greater than 0.4, the reconstruction error is smaller than 4% and the iteration number is smaller than 100.

#### 6.1.4. Conclusions

**Figure 9:**
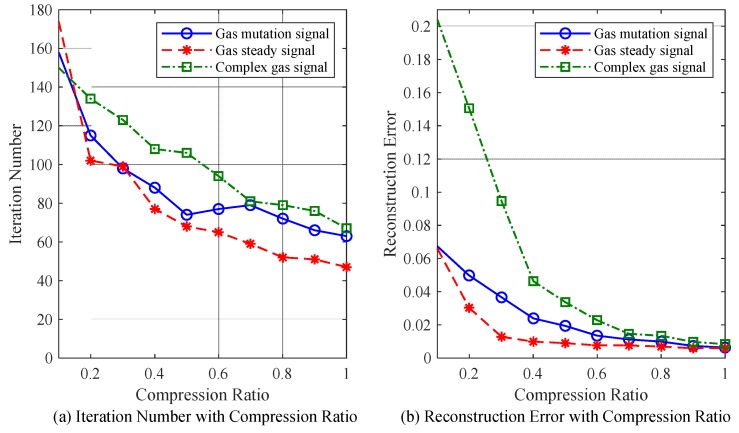
The relations of the compression ratio with the reconstruction error and iteration number for the reconstruction of three different gas signals using the TVS-MH algorithm.

Figure 9 illustrates the relationships of the compression ratio with reconstruction error and iteration number for the reconstruction of three different gas signals (namely the gas mutation signal, the gas steady signal and the complex gas signal) using the TVS-MH algorithm.

When the TVS-MH algorithm is used, the reconstruction for the gas steady signal exhibits the smallest iteration number and error, while the reconstruction for complex gas signal has the greatest iteration number and error. 

According to the above-described simulation results, the TVS-MH algorithm exhibits favorable applicability for different signals; under certain compression ratios, the reconstructions of the gas mutation signal, the gas steady signal and the complex gas signal can all satisfy the expectations, and the reconstruction accuracy can meet the requirements on the allowable error of a mine gas sensor; additionally, the computations are not too complex. All of these demonstrate that TVS-MH is applicable to compressing sampling of the mine gas signal.

### 6.2. Performance Analysis

In this section, the reconstruction performances and computation complexities using different signal reconstruction algorithms were compared. For each sparsity level *K* in the different measurements *M* and compression ratio *R*, we consider a Monte Carlo draw by repeating the same experiment 100 times for the same value of *K*, *M* or *R* [10].

#### 6.2.1. Relationships between the Reconstruction Error and the Compression Ratio for the Reconstruction of Different Gas Signals with Different Sparseness Degrees Using the TVS-MH Algorithm

**Figure 10:**
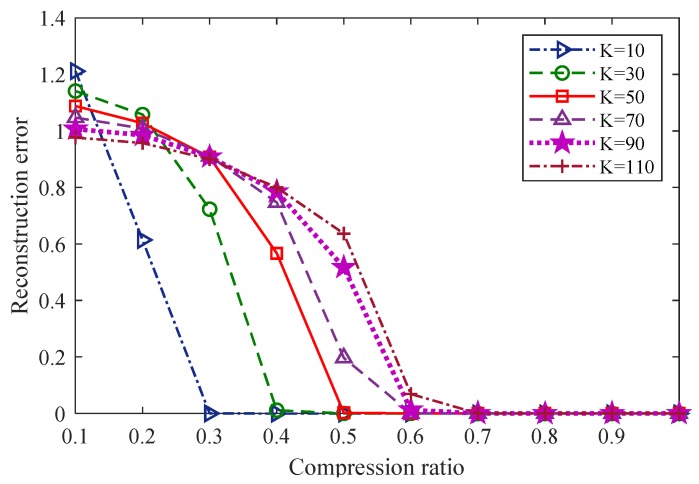
The relationship between the reconstruction error and the compression ratio for different gas signals with different sparseness degrees using the TVS-MH algorithm.

Figure 10 illustrates the relationship between the reconstruction error and the compression ratio for different gas signals with different sparseness degrees during which the signals were reconstructed using the TVS-MH algorithm and the signal length was 512 (*N* = 512). It can be observed that for K-sparse signals, the greater the value of K is, the higher the reconstruction error is.

#### 6.2.2. Reconfiguration Performance

**Figure 11:**
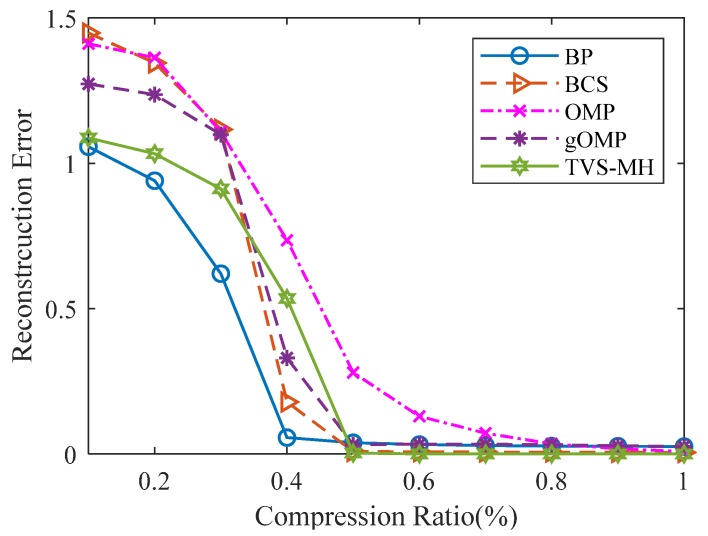
The relation between the reconstruction error and the compression ratio for the same sparse gas signal using different reconstruction algorithms.

Since the common sparse recovery algorithms can be classified into three main categories, Figure 11 illustrates the relationship between the reconstruction error and the compression ratio for the same sparse gas signal using different reconstruction algorithms, which are Convex and Relaxation (BP, TVS-MH), Greedy (OMP, gOMP), and Bayesian category (BCS). During the tests, a random signal with a sparseness of 50 and a length of 512 (*K* = 50 and *N* = 512) was used as the gas signal, and the program ran 100 times to take the average values.

It can be observed that among these five algorithms, the TVS-MH algorithm has more favorable reconstruction performances; in particular, TVS-MH algorithm exhibits steady performances in the test on CPU operation time, thereby suggesting its superiority.

#### 6.2.3. Algorithm Complexity

**Figure 12:**
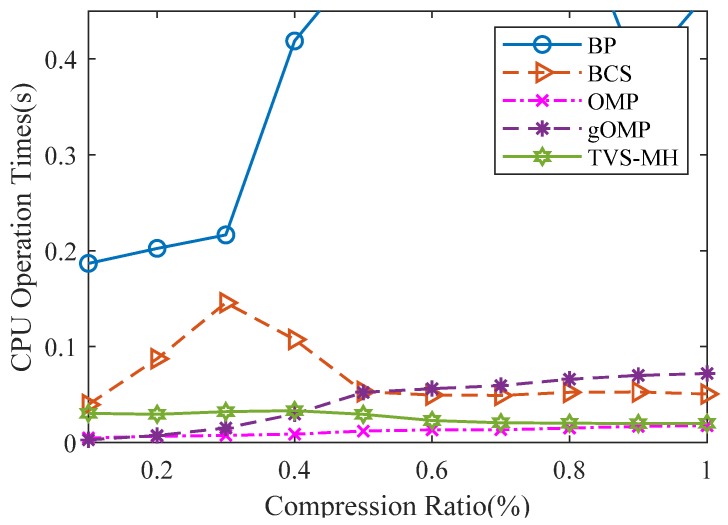
The relation between CPU operation time and compression ratio for a same sparse gas signal using different reconstruction algorithms.

The algorithm complexity can be expressed by program runtime. Figure 12 illustrates the relationship between CPU operation time and the compression ratio for the same sparse gas signal using different reconstruction algorithms. From Figure 12, the processing time and the recovery time of TVS-MH are observed as higher than those of OMP but lower than those of BP, BCS and gOMP. Table 4 presents the testing environment. 

## 7. Conclusions

With the development of coal mine IoT, a great number of intelligent miner’s lamps are put into use which has led to the multiplication of monitoring data. Data compression appears as an effective solution to high-efficiency transmission and storage. 

In view of the long linear structure of the coal mine roadway and the time sparsity of gas sensors, the paper studies the relationship between data transmission and compressed sensing in multi-hop networks and a Total Variation Sparsity based on Multi-Hop (TVS-MH) algorithm is proposed, which takes advantage of the time correlation of the mobile gas monitoring data in multi-hop networks and realizes the sparsity of monitoring data by introducing total variation.

The current study analyzed the sparseness characteristics of gas data, investigated the network model suitable for the transmission of gas data flow and used a pseudorandom measurement matrix to replace a random measurement matrix for performance improvement. Finally, through the intrusion of total variation of gas data, gas data were accurately and efficiently reconstructed using the TVS-MH algorithm. The present results indicate that high-efficiency acquisition and transmission of the measured data by mobile sensors in a distributed measurement network are possible.

## Figures and Tables

**Table 1:** The relations of *M* with the reconstruction error and the computation complexity during the reconstruction of gas mutation data.

*M*	51	102	154	205	256	307	358	410	461	512
Iteration number	158	115	98	88	74	77	79	72	66	63
Error (%)	6.74	4.98	3.66	2.39	1.94	1.35	1.12	0.99	0.73	0.63

**Table 2:** The relations of *M* with the reconstruction error and the computation complexity during the reconstruction of gas data under steady states.

*M*	51	102	154	205	256	307	358	410	461	512
Iteration number	174	102	99	77	68	65	59	52	51	47
Error (%)	6.61	3.03	1.28	0.99	0.90	0.76	0.76	0.69	0.59	0.60

**Table 3:** The relations of *M* with the reconstruction error and the computation complexity during the reconstruction of the complex gas signal.

*M*	43	86	129	172	215	258	301	354	397	430
Iteration number	157	127	121	107	97	87	79	80	78	70
Error (%)	21.02	14.27	7.87	5.37	3.06	2.50	1.71	1.09	1.04	0.89

**Table 4:** The testing environment.

Processor	Memory	Hard Disk	Operating System	Simulation Tool
Intel (R) Core (TM) i7-7700HQ CPU @2.80 GHz	16.0 GB	512 GB NVM	64-bit Windows 10 Home	MATLAB R2018a

## References

[1] Naranjo P.G.V., Pooranian Z., Shojafar M., Conti M., Buyya R. (2017). FOCAN: A Fog-supported Smart City Network Architecture for Management of Applications in the Internet of Everything Environments. arXiv.

[2] Mostafaei H., Shojafar M., Zaher B., Singhal M. (2017). Barrier coverage of WSNs with the imperialist competitive algorithm. J. Supercomput..

[3] Barrenetxea G., Ingelrest F., Schaefer G., Vetterli M., Couach O., Parlange M. SensorScope: Out-of-the-Box Environmental Monitoring. Proceedings of the 2008 International Conference on Information Processing in Sensor Networks.

[4] Yin H.-S. (2010). Gas Time Series Analytical Method and Its Early-Warning Application in Coalmine.

[5] Zhao Z.-K. (2012). Semi-Supervised Learning and Its Application to Coal Mine Gas Safety Information Processing.

[6] Qing-qing Huang Q.-Q., Tang B.-P., Deng L., Xiao X. (2014). Subband energy adaptive data compression method for wireless sensor networks. Chin. J. Sci. Instrum..

[7] Xie Z.-J., Wang L., Lin Y., Liu Y. (2006). An Algorithm of Data Aggregation Based on Data Compression for Sensor Networks. J. Softw..

[8] Ziv J., Lempel A. (2003). A universal algorithm for sequential data compression. IEEE Trans. Inf. Theory.

[9] Li S.-T., Wei D. (2009). A Survey on Compressive Sensing. Acta Autom. Sin..

[10] Arjoune Y., Kaabouch N., El Ghazi H., Tamtaoui A. Compressive sensing: Performance comparison of sparse recovery algorithms. Proceedings of the 2017 IEEE 7th Annual Computing and Communication Workshop and Conference.

[11] Xue T., Dong X., Shi Y. (2013). Multiple Access and Data Reconstruction in Wireless Sensor Networks Based on Compressed Sensing. IEEE Trans. Wirel. Commun..

[12] Uthayakumar J., Vengattaraman T., Amudhavel J. (2017). Data compression algorithm to maximize network lifetime in wireless sensor networks. J. Adv. Res. Dyn. Control. Syst..

[13] Zou Z., Bao Y., Li H., Spencer B.F., Ou J. (2015). Embedding Compressive Sensing-Based Data Loss Recovery Algorithm Into Wireless Smart Sensors for Structural Health Monitoring. IEEE Sens. J..

[14] Baraniuk R. (2007). Compressive sensing. IEEE Signal Process. Mag..

[15] Mallat S. (1996). A Wavelet Tour of Signal Processing.

[16] Candès E.J., Donoho D.L. (2000). Curvelets: A Surprisingly Effective Nonadaptive Representation for Objects with Edges.

[17] Sun Y.-B., Xiao L., Wei Z.-H. (2008). Sparse Representations of Images by a Multi-component Gabor Perception Dictionary. Acta Autom. Sin..

[18] Aharon M., Elad M., Bruckstein A.M. (2006). The K-SVD: An algorithm for designing of overcomplete dictionaries for sparse representations. IEEE Trans. Image Process..

[19] Rauhut H., Schnass K., Vandergheynst P. (2008). Compressed sensing and redundant dictionaries. IEEE Trans. Inf. Theory.

[20] Candès E.J., Tao T. (2005). Decoding by linear programming. IEEE Trans. Inf. Theory.

[21] Candès E.J., Romberg J., Tao T. (2006). Stable signal recovery from incomplete and inaccurate measurements. Commun. Pure Appl. Math..

[22] Candès E.J., Tao T. (2006). Near optimal signal recovery from random projections: Universal encoding strategies. IEEE Trans. Inf. Theory.

[23] Hayashi K., Nagahara M., Tanaka T. (2013). A user’s guide to compressed sensing for communications systems. IEICE Trans. Commun..

[24] Haupt J., Bajwa W.U., Rabbat M., Nowak R. (2008). Compressed sensing for networked data. IEEE Signal Process. Mag..

[25] Lee S., Pattem S., Sathiamoorthy M., Krishnamachari B., Ortega A. (2009). Compressed Sensing and Routing in Multi-Hop Networks.

[26] Arjoune Y., Kaabouch N., El Ghazi H., Tamtaoui A. (2018). A performance comparison of measurement matrices in compressive sensing. Int. J. Commun. Syst..

